# Declaration of Independence

**Published:** 1860-01

**Authors:** 


### DECLARATION OF INDEPENDENCE.

"When in the course of human events—" Hold! We don't mean *the* declaration, but *a* declaration, as follows:

"It is the design of the editorial corps to make it a thoroughly independent, professional Quarterly Review. In order to do this it will be conducted as experience has shown it best to conduct such publications.

"That these, gentlemen editors, occupy an independent position, it is enough to say that they have no merchandise to dispose of, no books to introduce to the profession, no tickets to sell. And that they may not be open to the suspicion that this work may be made to accrue through outside influence or reputation, to the benefit of their practice, they prefer that their names do not appear."

The above is from the prospectus for the third volume of the "American Dental Review." We are glad that Dr. Leslie is to be somewhat relieved in his labors ; but, we are at a loss to know what call there was for some of the statements of this declaration. For example, " It is the design of the editorial corps to *make* it a thoroughly independent Quarterly Review." Has it not been " thoroughly independent" under the exclusive management of its publisher and late editor ? It would appear not, for otherwise, it would be announced that it is the design to *continue* it as a thoroughly independent Review.

To enable the "gentleman editors" to be independent, " they have no merchandise to dispose of, no books to introduce to the profession, no tickets to sell." Ah ! that explains it ! The reason that it is now necessary to *make* the " Review" independent is that its late editor had merchandise to dispose of," " books (of foil) to introduce to the profession," and once had " tickets to sell," consequently he did not "occupy an independent position." Are we to understand the publisher that these "gentlemen editors" could not be independent if they had merchandise to dispose of, books to introduce, or tickets to sell ? If so it is well that they are free from these incumbrances ; for stumbling blocks should be kept out of the way of the weak. But if the publisher of the " Register" ever pays its editors such a left-handed compliment, one of them will, most likely, resign.

But these " gentlemen editors " are to be not only independent, but above, or at least " not open" to suspicion, hence, " they prefer that their names do not appear." All right, certainly, but when John Hancock made a declaration of *independence* he put his name to it. But these gentlemen seem fearful, on account of their great ability, perhaps, that their editorial reputation " may be made to accrue to the benefit of their practice." This would be sad indeed, but as they "occupy an independent position," they would, probably, survive the catastrophe, and live to realize that the chastening was for their good. Their position seems to be really independent in one respect ; if their editorial labors prove a failure, they can hold quiet and their practice will not likely suffer ; but if they prove a success, they can tell some friend, as a profound secret, that they are the "gentlemen editors," and poor mystery-loving humanity will be all agog.

We thought the "Review" was "thoroughly independent" before, but, if the publisher thinks differently, we have no disposition to quarrel with him. It is a mere matter of opinion, and the profession will believe the one most likely to be in posession of all the facts. But we differ with the publisher about the character of his editors, and here we are not so ready to yield the point. We believe they would be independent, even if they had "tickets to sell." If they would not, independence has nothing to hope for from them. Fluid mercury might be forced into a central cavity of an upper molar, with a hair broach, or cambric needle ; but, by no contrivance can independence be forced into a man who cannot manifest it over his own signature, in spite of such paltry considerations as the publisher refers to. W.

The American Dental Review suggests carbolic acid as a good application where a "wound is slow to heal." The prescription is a good one in many cases, but by no means new. It might be conveniently applied with the "serrated petugar," mentioned in page 160 of the November number of the "Review." W.

